# Supplementary material for: Investigation on the Mass Distribution and Chemical Compositions of Various Ionic Liquids-Extracted Coal Fragments and Their Effects on the Electrochemical Performance of Coal-Derived Carbon Nanofibers (CCNFs)
Source: Nanomaterials (Basel). 2021 Mar 8;11(3):664. doi: 10.3390/nano11030664 (PMC8000264; doi:10.3390/nano11030664)
Supplement: Supplementary file 1 [file nanomaterials-11-00664-s001.pdf]

nanomaterials

MDPI

Supplementary Materials: Investigation on the Mass Distribution and Chemical Compositions of Various Ionic Liquids-Extracted Coal Fragments and their Effects on the Electrochemical Performance of Coal-Derived Carbon Nanofibers (CCNFs)

Shuai Tan [1], Theodore John Kraus [2], Mitchell Ross Helling [2], Rudolph Kurtzer Mignon [2], Franco Basile [2] and Katie Dongmei Li-Oakey [1,*]

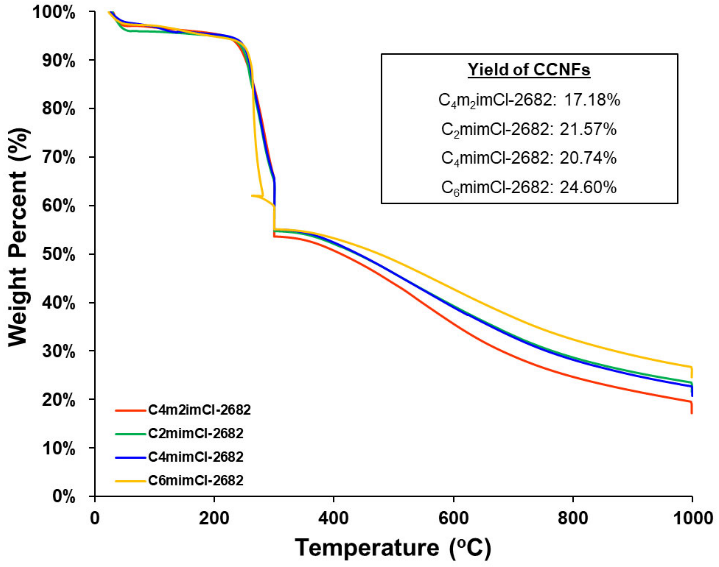

Figure S1. The TGA profile of CCNFs fabrication process through oxidation and carbonization. The inset is the calculated yields of each CCNFs from the precursors.

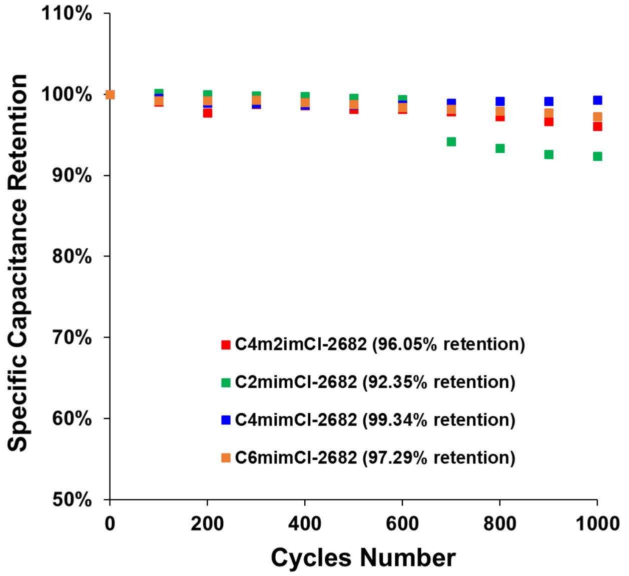

Figure S2. The specific capacitance retention of each CCNF electrode during 1000 cycles of GCD at the current density of 1A/g.

Table S1. Summary of SAD-AD analysis on ILs-extracted coal precursors.

Coal Precursor	Saturates (%)	Aromatics (%)	Resins (%)	Asphaltenes (%)
C_4m_2imCl-2682	21.58	46.89	22.77	6.58
C_2mimCl-2682	6.05	69.68	14.62	8.51
C_4mimCl-2682	14.86	58.04	19.20	6.50
C_6mimCl-2682	10.96	62.40	19.08	6.13
C_6mimCl-2684	3.19	56.38	14.42	25.09

Table S2. Summary of GC-MS analysis on C_2mimCl-2682 coal extract with corresponding retention time.

	**C_2mimCl-2682 Coal Extract**
Retention Time (mins)	Tent. ID
5.94	methylamine (2-TMS derivative)
6.403	ethylene glycol (2-TMS derivative)
7.655	carbodiimide (2-TMS derivative)
8.251	boric acid (3-TMS derivative)
9.601	lactic acid (2-TMS derivative)
10.018	acetamide (TMS-derivative)
10.343	1-(3-methylbutyl)-2,3,4,6-tetramethylbenzene
29.528	phthalate (plasticizer contaminant)
Several unidentified components (10)	
29 components, 19 targets	

Table S3. Summary of GC-MS analysis on C_4mimCl-2682 coal precursor with corresponding retention time.

	**C_4mimCl-2682 Coal Precursor**
Retention Time (mins)	Tent. ID
5.939	methylamine (2-TMS derivative)
6.366	ethylene glycol (2-TMS derivative)
8.246	boric acid (3-TMS derivative)
8.615	4-methoxy-1-butanol (TMS derivative)

9.594	lactic acid (2-TMS derivative)
11.493	1,4-butanediol (2-TMS derivative)
Several unidentified components (8)	
26 components, 20 targets	

Table S4. Summary of GC-MS analysis on C_6mimCl-2682 coal precursor with corresponding retention time.

	**C_6mim-2682 Coal Precursor**
Retention Time (mins)	Tent. ID
5.94	methylamine (2-TMS derivative)
6.013	n,n-dimethyloctylamine
6.365	ethylene glycol (2-TMS derivative)
8.248	boric acid (3-TMS derivative)
9.596	lactic acid (2-TMS derivative)
10.342	1-(3-methylbutyl)-2,3,4,6-tetramethylbenzene
Several unidentified components (9)	
19 components, 10 targets	

Table S5. Summary of GC-MS analysis on C_4m_2imCl-2682 coal precursor with corresponding retention time.

	**C_4m_2im-2682 Coal Precursor**
Retention Time (mins)	Tent. ID
6.03	methylamine (2-TMS derivative)
6.365	ethylene glycol (2-TMS derivative)
7.655	carbodiimide (2-TMS derivative)
8.255	boric acid (3-TMS derivative)
9.601	lactic acid (2-TMS derivative)
10.347	1-(3-methylbutyl)-2,3,4,6-tetramethylbenzene
11.466	sulfuric acid (2-TMS derivative)
29.537	phthalate (plasticizer contaminant)
Several unidentified components (9)	
24 components, 15 targets	

Table S6. Electrochemical performance comparison of CCNFs derived from different coal extracts.

**Electrode Materials**	**Specific Capacitance (F/g)**	**Current Density (A/g)**	**Power Density (kW/kg)**	**Energy Density (Wh/kg)**	**References**
CCNFs from Acid-wash coal Char	210	1	-	-	[1]
CCNFs from Oxidized Coal Char	260	1	-	-	[2]
Nitrogen-doped CCNFs from Asphaltene	301	1	0.25	8	[3]
CCNFs from CO_2 Supercritical-derived Coal Tar	409	0.5	2.75	7.04	[4]
CCNFs from Solar Pyrolysis of Pinewood	349	0.5	5	5.11	[5]
CCNFs from C_6mimCl Coal Extract	295	1	0.6/12	21.1/7.6	This study

References

1. Zhao, H.Y.; Wang, L.X.; Jia, D.Z.; Xia, W.; Li, J.; Guo, Z.P. Coal Based Activated Carbon Nanofibers Prepared by Electrospinning. *J. Mater. Chem. A* **2014**, *2*, 9338–9344.
2. He, Y.; Wang, L.; Jia, D. Coal/PAN Interconnected Carbon Nanofibers with Excellent Energy Storage Performance and Electrical Conductivity. *Electrochim. Acta* **2016**, *194*, 239–245.
3. Ni, G.; Qin, F.; Guo, Z.; Wang, J.; Shen, W. Nitrogen-Doped Asphaltene-Based Porous Carbon Fibers as Supercapacitor Electrode Material with High Specific Capacitance. *Electrochim. Acta* **2020**, *330*, 135270.
4. Wang, T.; He, X.; Gong, W.; Sun, K.; Lu, W.; Yao, Y.; Chen, Z.; Sun, T.; Fan, M. Flexible Carbon Nanofibers for High-Performance Free-Standing Supercapacitor Electrodes Derived from Powder River Basin Coal. *Fuel* **2020**, *278*, 117985.
5. Wang, T.; Rony, A. H.; Sun, K.; Gong, W.; He, X.; Lu, W.; Tang, M.; Ye, R.; Yu, J.; Kang, L.; Luo, H.; Smith, S. J.; Eddings, E. G.; Fan, M. Carbon Nanofibers Prepared from Solar Pyrolysis of Pinewood as Binder-Free Electrodes for Flexible Supercapacitors. *Cell Reports Phys. Sci.* **2020**, *1*, 100079.
